# Adverse events following immunization of COVID-19 vaccine among children aged 6–11 years

**DOI:** 10.3389/fpubh.2022.999354

**Published:** 2022-10-25

**Authors:** Fitriana Puspitarani, Mei Neni Sitaresmi, Riris Andono Ahmad

**Affiliations:** ^1^Department of Biostatistics, Epidemiology, and Population Health, Faculty of Medicine, Public Health, and Nursing, Gadjah Mada University, Yogyakarta, Indonesia; ^2^Departement of Child Health, Faculty of Medicine, Public Health, and Nursing, Gadjah Mada University, DR. Sardjito Hospital, Yogyakarta, Indonesia; ^3^Center for Tropical Medicine, Faculty of Medicine, Public Health, and Nursing, Gadjah Mada University, Yogyakarta, Indonesia

**Keywords:** AEFI, COVID-19 vaccine, children, prevalence, determinant factors

## Abstract

**Introduction:**

Starting in December 2021, the Indonesian Government has recommended inactivated SARS-CoV-2 vaccine (CoronaVac) for children aged 6–11 years. This study aims to determine the prevalence and determinant factors of adverse events following immunization (AEFI) of the first dose and the second dose of the COVID-19 vaccine among children aged 6–11 years old.

**Materials and methods:**

We conducted a cross-sectional study in Bantul District, Yogyakarta, Indonesia, in February–March 2022. Data were collected by trained interviews with 1,093 parents of children 6–11 years old who received the first dose and the second dose of the COVID-19 vaccine. Data were analyzed with chi-square and logistic regression.

**Results:**

The prevalence of AEFI in the first dose of the COVID-19 vaccine was 16.7%, while the second dose was 22.6%. The most common symptoms of AEFI at the first dose were local site pain and fever, while at the second dose were cough and cold. Determinants of AEFI of COVID-19 vaccination among children were girls with OR 1.31 (95% CI 1.0–1.7; *P* 0.04), mass-setting of vaccination with OR 0.70 (95% CI 0.5–0.9; *P* 0.01), the history of AEFI in childhood vaccination with OR 1.63 (95% CI 1.2–2.2; *P* < 0.01) and administering other vaccines within 1 month before COVID-19 vaccination, with OR 5.10 (95% CI 2.1–12.3 *P* < 0.01).

**Conclusion:**

The prevalence of AEFI in the first and the second dose of inactivated COVID-19 vaccine was comparable to that reported in the clinical trial study and the communities. Risk communication should be provided to the child and their parents regarding the risk of mild AEFI of the COVID-19 vaccine, especially for children with a history of AEFI in childhood vaccination and who received other vaccines containing the same adjuvant with CoronaVac within 1 month. A mass-setting of vaccination should be taken as an advantage to educate parents about the risk of AEFI and also about the reporting pathways.

## Introduction

Coronavirus 2019 (COVID-19) is a newly emerging disease and was announced as a global pandemic on March 2020 (1). Confirmed cases of COVID-19 have been reported by almost all countries globally, including Indonesia. Until early June 2022, this disease has infected more than 550 million people worldwide and caused more than five million deaths (2). While in Indonesia, more than 6 million people have been infected, with more than 150,000 deaths (3).

Vaccination against Coronavirus Disease (COVID-19) is one of the efforts taken to accelerate the occurrence of herd immunity and break the chain of transmission of COVID-19 (4). Nowadays, Indonesia has used 10 brands of COVID-19 vaccine designated for 208,265,720 civilians divided into four steps until March 2022 (5). At the end of 2021, vaccination coverage in Indonesia reached 77.3% for dose one and 54.6% for dose two (6).

Compared to adults, children and adolescents infected with SARS-CoV-2 are more likely to be asymptomatic or have milder symptoms with a lower risk of mortality (7), especially because children aged 6–11 years are in the process of alveologenesis and microvascular (8). However, those with underlying health comorbidities might be at risk for severe COVID-19, such as the multisystem inflammatory syndrome (9). In addition, children and adolescents can be important transmitters of SARS-CoV-2 in communities. Therefore, including children in the implementation of COVID-19 vaccination may give indirect benefits through community protection or herd immunity (7).

Starting in December 2021, the Indonesian Government has recommended the COVID-19 vaccine for children aged 6–11 years (10). Children aged 6–11 years are one of the targeted people to get vaccinated using one type of vaccine, namely CoronaVac, which is an inactivated virus vaccine developed by Sinovac Life Sciences (Beijing, China) by injecting intramuscularly in the upper arm at a dose of 0.5 ml (11) with two doses of 28 days intervals between doses (12). In the double-blind, randomized, controlled, phase 1/2 clinical trial, the CoronaVac was well tolerated and safe and induced humoral responses in children and adolescents aged 3–17 years (13).

In the Special Region of Yogyakarta by 30 June 2022, Bantul District was the second-largest contributor of COVID-19 cases with a total of 68,625 cases with 67,111 recovered (97.8%) and 1,506 deaths (2.2%) (14). A screening survey of COVID-19 in school settings in the Bantul District showed that the prevalence of COVID-19 infection was 4.2%, with unvaccinated status at risk of being infected in schools (15).

Along with increasing immunization coverage, there are also adverse events following immunization (AEFI), which is an untoward medical occurrence that follows vaccination and does not necessarily have a causal relationship with vaccine usage (16). There is not all kind of AEFI that is only vaccine-related but also anxiety-related due to immunization stress reaction, accidental, or procedural error (17). AEFI problems are closely related to public perception about the efficacy and safety of the vaccine. This is one of the factors related to the community's decision to accept or reject the vaccine. Vaccine refusal contributes to reduced vaccine coverage and herd immunity, leading to centralized outbreaks or pockets of infection in a specific group (18).

The National Agency of Drug and Food Control of Indonesia has issued an Emergency Use Authorization (EUA) based on studies of clinical trials phases 1, 2, and 3 on the safety and efficacy of the COVID-19 vaccine (19). From the clinical trial phase 1/2 CoronaVac showed a good safety profile and immunogenicity in children aged 3–17 years, the prevalence of adverse events was 27%, and most of them were mild and moderate in severity (13).

Du et al. conducted a systematic review and meta-analysis, which included six randomized controlled trials: three mRNA vaccines, two inactivated vaccines, and one adenoviral vector vaccine, assessing the safety, immunogenicity, and efficacy of the COVID-19 vaccine in children aged 3–17 years old. The study found that compared with mRNA vaccines and adenovirus vector vaccines, inactivated vaccines have a more satisfactory safety profile, both after initial and booster doses (20). As a new vaccine, it is necessary to know its security once implemented in a broader population. In addition, assessing the risk factors of AEFI in COVID-19 vaccination for children aged 6–11 years is essential. The study aims to assess the prevalence of inactivated COVID-19 vaccine AEFI and its determinants in children aged 6–11 years.

## Materials and methods

We conducted a cross-sectional study in Bantul District, Yogyakarta, Indonesia, in February 2022. Before the study was conducted in early February 2022, vaccination coverage for children aged 6–11 years in Bantul District reached >90% for dose one. To achieve high coverage, vaccination is given in schools or the public service area. In total, 74.982 children aged 6–11 years were vaccinated during the vaccination drive. The first dose was given from December 2021 to January 2022. The second dose was administered after 28 days after the first dose. All recipients are routinely monitored at study sites for 15–30 min post-vaccine administration as part of the standard operating procedure for vaccination (12). Before administering a vaccine, there is a screening process to gather information about flu-like symptoms 7 days before and history of COVID-19 infection as well a history of close contact with a COVID-19 case. Vaccination officers can decide not to vaccinate children who have flu-like symptoms, are in close contact, or have a history of COVID-19 infection within a certain agreed time (11).

The target population was children aged 6–11 years who received the inactivated COVID-19 vaccine (CoronoVac) in Bantul District, which is 74,982 (21). Samples were those who met inclusion criteria (who received the second dose of the COVID-19 vaccine) and were obtained with stratified random sampling by clustering schools or other vaccination sites based on their regional characteristics (rural and urban). A cluster sample was taken from each list of vaccination sites using an MS Excel 365 random number generator.

We used an XLSForm from Microsoft Excel to develop the questionnaire and then upload it to the KoboToolbox, an electronic questionnaire developed by GitHub, Inc (22). All data collection can be taken online or offline, but a network connection is required to upload finalized forms. When all data collection has finished, then we export and download the final data into XLS format and enter it into Microsoft Excel for the cleaning and coding process before importing it to STATA 16. We use limiting parameters such as respondents' age must be at least 20 and set up a conditional question to minimize error in the data entry process. Enumerators could access the e-questionnaire through a web link, but KoboToolbox requires a username and password for accessing data and managing the data. While electronic forms have risks to ensure their reliability, validation is done by telephone when they find an input error so that respondents will be asked to provide their mobile number.

The questionnaire was developed based on the AEFIs standard set by the Ministry of Health of Indonesia (11) and consists of demographic characteristics (age, gender, parents' education level, parents' occupational, residency, school strata, and vaccination place), medical anamnesis (history of AEFI, comorbidities, history of post-confirmed COVID-19), vaccine-related anamnesis (history of administering another vaccine 1 month before) and any post-vaccine-related symptoms or AEFI by a recall for 14 days after receiving the second dose.

We define parents' educational level as elementary to junior high school (± 9 years of study) and senior high school to higher education (>9 years of study). Vaccination place was defined as limited settings, which are limited space in a homogeneous situation, such as school and public health center buildings, and mass-settings, which are open spaces in heterogeneous situations, such as village hall, park, or other multipurpose building. Comorbidities were defined as chronic diseases under treatment (11).

The questionnaire was piloted among 48 parents who have children aged 6–11 years and were not part of the sample. Trained enumerators collected data from the caregivers while waiting for their children to receive the second dose of the COVID-19 vaccine. The person-to-person interviews aim to recall AEFI in the first dose, then the caregiver will be asked for permission to conduct another phone-based interview 14 days later to follow up on AEFI of the second dose. The respondents are parents/guardians of children aged 6–11 years who received the COVID-19 vaccination in the Bantul District.

The minimal sample size required for the study was estimated to be 455 per cluster [urban and rural (23)], anticipating that 18.1% of study subjects will have AEFI with a 5% level of significance, 5% absolute error margin at a 95% confidence interval, and non-response estimates due to refusal or loss to follow up by 20%, so the minimum sample size was equal to 1,092 children and their parents/guardians. The inclusion criteria for this study were children aged 6–11 years who were accompanied by their parents/guardians who lived in Bantul District and had received vaccination in Bantul District. The exclusion criteria were incomplete information.

**Figure 1:**
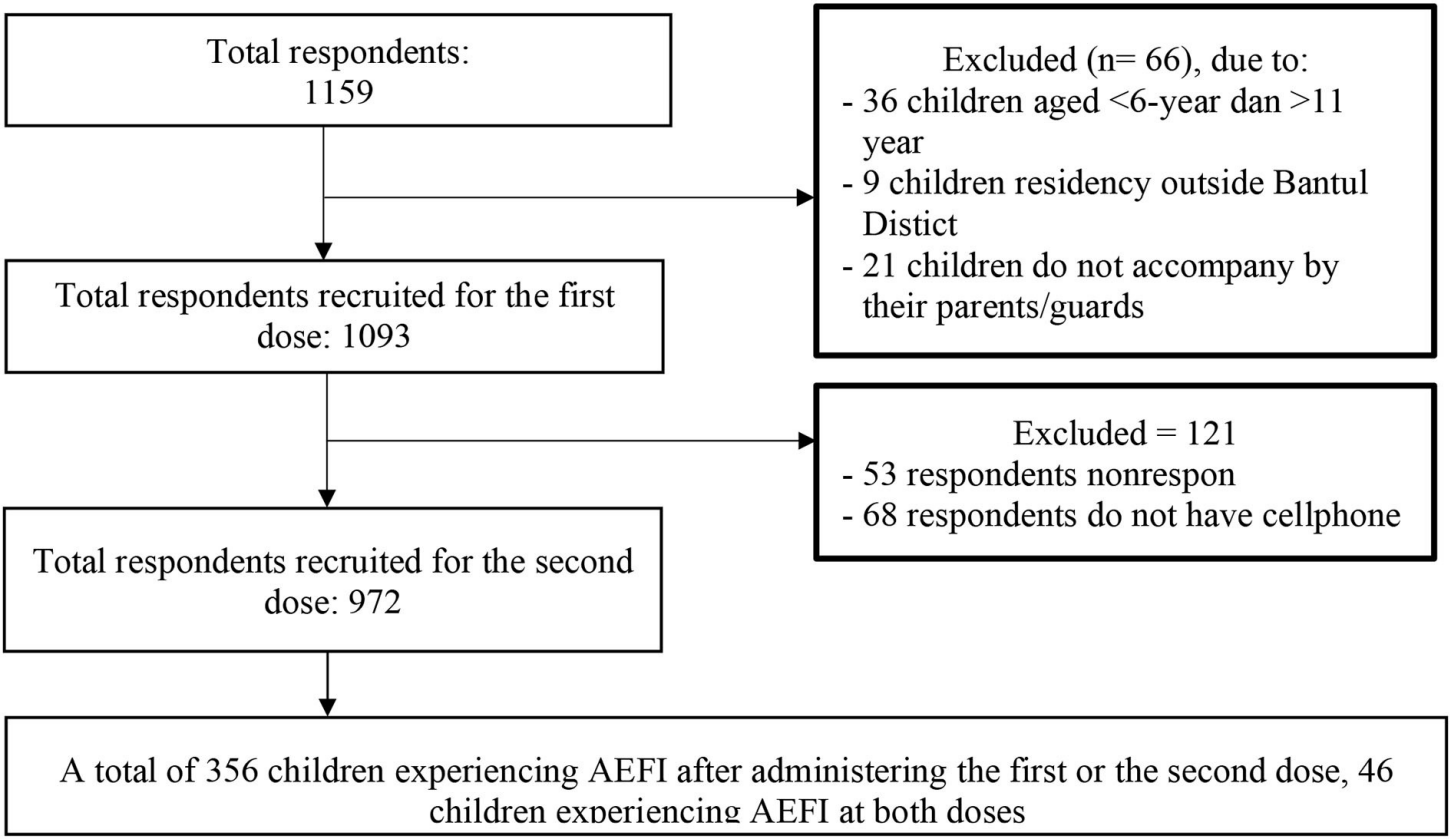
Respondents recruitment process.

The data analysis was carried out on 1,093 subjects for the first dose and 972 subjects for the second dose (Figure 1). AEFI events as a dependent factor will be considered once for every child, so whenever children experience AEFI at both doses of CoronaVac, it will be counted as one event. The data was analyzed statistically by STATA 16 using the chi-square test and logistic regression. Variables with a *P* < 0.25 was continued into the multivariate analysis and considered significant if the *P* < 0.05.

Ethical approval was obtained from the Ethical Committee Faculty of Medicine, Public Health, and Nursing ethics committee, Universitas Gadjah Mada, with registration number KE/FK/0112/EC/2022. Written informed consent was obtained from the parents as respondents represent their children. Participation was voluntary, and confidentiality was ensured.

## Results

Among 1,159 respondents who met the inclusion criteria, 66 (6%) respondents were excluded: 36 children (3%) due to an age of < 6 years or more than 11 years. Nine children (1%) lived outside Bantul District and 21 children (2%) were not accompanied by their parents. A total of 1,093 children were recruited. A phone-based interview was conducted 14 days after the second dose, 121 respondents must be excluded due to being unresponsive while being contacted by enumerators nor do not have a cellphone (Figure 1). Based on Table 1, most respondents (79.4%) were mothers, and mostly had senior high school or higher education levels (71.1%). The most frequent occupation of the respondents was in the informal sector (82.9%).

**Table 1:** Characteristics respondents and children.

**Variable**	**Categories**	***n* (%)**
**Respondents**		
Relation with children	Father	173 (15.8)
	Mother	870 (79.4)
	Other	50 (4.6)
Gender	Male	183 (16.7)
	Female	910 (83.3)
Age (year)	Early adulthood (20–40)	730 (66.8)
	Middle adulthood (41–60)	356 (32.6)
	Late adulthood (>60)	7 (0.6)
Education	Elementary-junior high school	316 (28.9)
	Senior high school-higher education	777 (71.1)
Occupation	Informal	906 (82.9)
	Formal	187 (17.1)
Residency	Urban	480 (43.9)
	Rural	613 (56.1)
Vaccination site	Limited setting	726 (66.4)
	Mass setting	267 (33.6)
**Children**		
Gender	Male	561 (51.3)
	Female	531 (48.7)
Age (years)	6	323 (21.2)
	7	207 (18.9)
	8	219 (20.0)
	9	162 (14.8)
	10	146 (13.4)
	11	127 (11.6)
School status	Public	770 (70.4)
	Private	323 (29.6)
History of AEFI in childhood	Yes	249 (22.8)
	No	844 (77.2)
Comorbidities	Yes	25 (2.6)
	No	1,065 (97.4)
History of COVID-19	Yes	43 (3.9)
	No	1,050 (96.7)
Administering other vaccines within 1 month before	Yes	28 (2.6)
	No	1,065 (97.4)

Five hundred and sixty-one out of 1,093 (51.3%) children were male, with an average age of 8 years. Most children did not have comorbidities (97.4%) and did not receive any other vaccinations within 1 month before receiving the COVID-19 vaccination (97.4%). Most children did not have a history of AEFI in childhood vaccination (77.2%). Before children were scheduled to receive COVID-19 vaccination, 3.9% of children had a COVID-19 diagnosis (Table 1).

**Figure 2:**
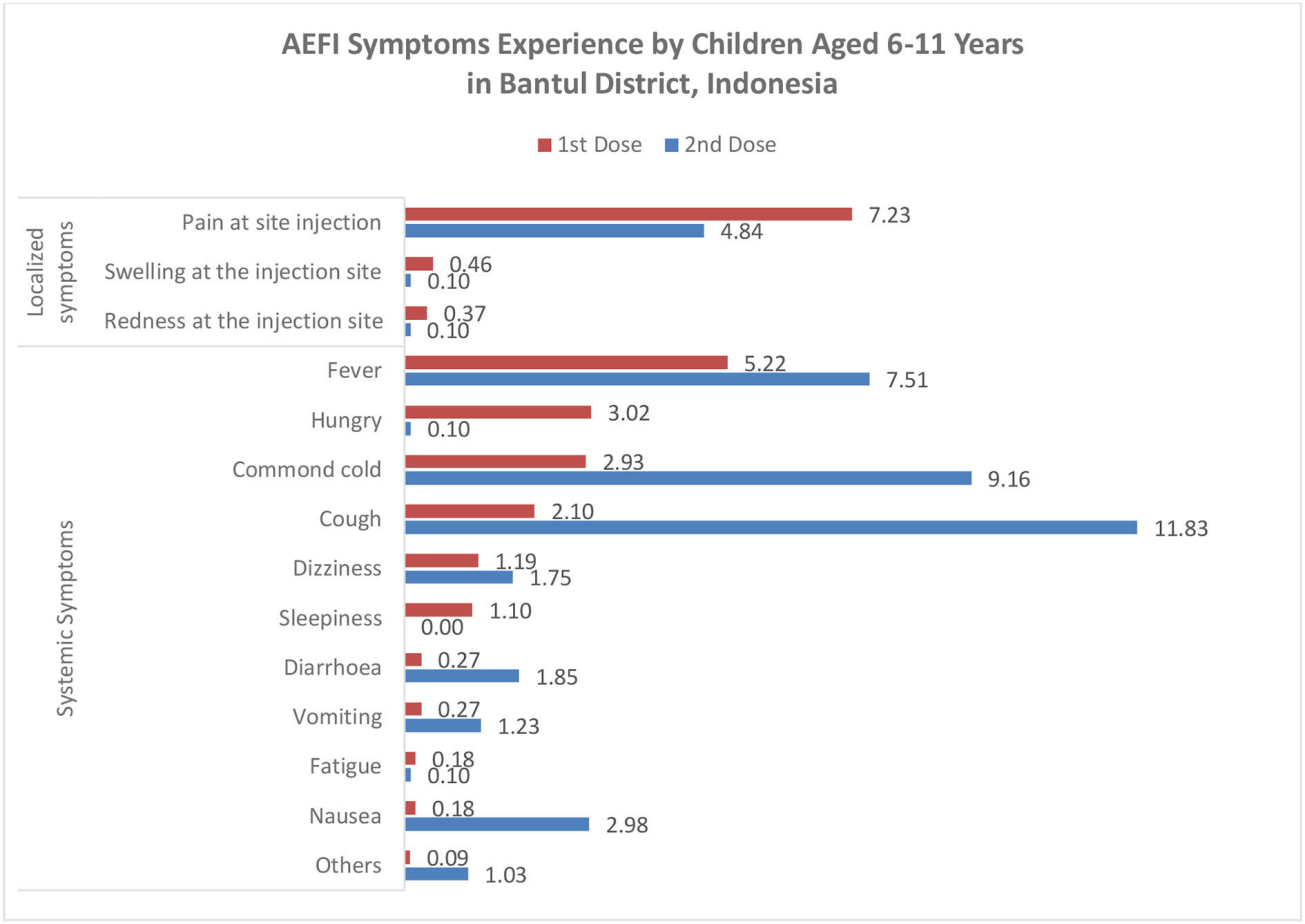
AEFI symptoms experiencing by children aged 6–11 years.

We found that 182 out of 1,093 (16.7%) children reported AEFI after the first dose, 220 out of 972 (22.6%) children reported AEFI after the second dose, and 46 children experienced AEFI after the first and second doses. All symptoms were considered mild to moderate. In the first dose, most symptoms were local reactions such as pain at the injection site (7.2%), and systemic responses such as fever (5.2%), while in the second dose, most symptoms were systemic symptoms such as cough (11.8%) and common cold (9.2%) (Figure 2). A total of 18 (9.9%) and 35 (15.9%) children visited health providers to get treatment due to their AEFI. We found several rare symptoms such as hungry (3.0%) and sleepiness (1.1%) in the first dose. The symptoms of AEFIs in both doses are commonly observed on the same day of the vaccination and last mostly until the fourth day after that (Table 2).

**Table 2:** Time observed of onset and duration of adverse event following the first and the second dose of CoronaVac vaccine for children aged 6–11 years.

**Day observed**	**First dose**		**Second dose**	
	**Onset *n* (%)**	**Duration *n* (%)**	**Onset *n* (%)**	**Duration *n* (%)**
0	144 (79.1)	86 (47.3)	73 (33.2)	12 (5.5)
1	15 (8.2)	56 (30.8)	26 (11.8)	32 (14.5)
2	2 (1.1)	26 (14.3)	16 (7.3)	49 (22.3)
3	1 (0.5)	5 (2.7)	8 (3.6)	35 (15.9)
4	1 (0.5)	3 (1.6)	11 (5.0)	45 (20.5)
5	–	3 (1.6)	10 (4.6)	14 (6.4)
6	–	1 (0.5)	18 (8.2)	11 (5.0)
7	6 (3.3)	2 (1.1)	20 (9.1)	6 (2.7)
8	1 (0.5)	–	8 (3.6)	3 (1.4)
9	–	–	13 (5.9)	4 (1.8)
10	–	–	1 (0.5)	0 (0.0)
11	–	–	3 (1.4)	1 (0.5)
12	–	–	4 (1.8)	0 (0.0)
13	1 (0.5)	–	3 (1.4)	2 (0.9)
14	2 (1.1)	–	3 (1.4)	6 (2.7)
>14	9 (4.9)	–	3 (1.4)	

Girls, mass-setting for the place of vaccination, having a history of AEFI in childhood vaccination, and administering another vaccine within 1 month have a significantly higher risk of AEFI of the COVID-19 vaccine in children aged 6–11 years (Table 3).

**Table 3:** Determinants of adverse events following of CoronaVac vaccine for children aged 6–11 years.

**Variable**	**AEFI**	**No AEFI**		**Univariate**			**Multivariate**	
	***n* = 356 (%)**	***n* (%)**	**OR**	**95% CI**	***P*-value**	**OR**	**95% CI**	***P*-value**
**Gender**								
Male (561)	166 (29.6)	395 (70.4)	1	–	–	1	–	–
Female (532)	190 (35.7)	342 (64.3)	1.32	1.0–1.7	0.03*	1.31	1.0–1.7	0.04*
**Age group**								
6–8 years (658)	219 (33.3)	439 (66.7)	1.08	0.8–1.4	0.54			
9–11 years (435)	137 (31.5)	298 (68.5)	1	–	–			
**School stratum**								
Public (770)	253 (32.8)	517 (67.1)	1	–	–			
Private (323)	103 (31.9)	220 (68.1)	0.96	0.7–1.3	0.75			
**Residency**								
Urban (480)	158 (32.9)	322 (67.1)	1	–	–			
Rural (613)	198 (32.3)	415 (67.7)	0.97	0.7–1.3	0.83			
**Parent's educational level**								
Elementary (316)	92 (29.1)	224 (70.9)	0.79	0.6–1.1	0.12	0.82	0.6–1.1	0.17
Senior and higher (777)	264 (34.0)	513 (66.0)	1	–	–	1	–	–
**Parent's occupational**								
Informal (906)	290 (32.0)	616 (68.0)	0.86	0.6–1.2	0.38			
Formal (187)	66 (35.3)	121 (64.7)	1	–	–			
**Vaccination took place**								
Limited setting (739)	257 (34.8)	482 (65.2)	1	–	–	1	–	–
Mass setting (354)	99 (278.0)	255 (72.0)	0.73	0.5–0.9	0.03*	0.70	0.5–0.9	0.01*
**History of AEFIs in childhood vaccination**								
Yes (249)	105 (42.2)	144 (57.8)	1.72	1.3–2.3	< 0.01*	1.63	1.2–2.2	< 0.01*
No (844)	251 (29.7)	593 (70.3)	1	–	–	1	–	–
**Comorbidities**								
Yes (25)	11 (44.0)	14 (56.0)	1.65	0.7–3.9	0.22	1.25	0.5–2.9	0.59
No (1,068)	345 (32.3)	723 (67.7)	1	–	–			
**Administering other vaccines**								
Yes (28)	21 (75.0)	7 (25.0)	6.5	2.6–18.3	< 0.01*	5.10	2.1–12.3	< 0.01*
No (1,065)	335 (31.5)	730 (68.5)				1	–	–
**History of COVID-19**								
Yes (43)	13 (30.2)	30 (69.8)	0.89	0.4–1.8	0.74			
No (1,050)	343 (32.7)	707 (67.3)	1	–	–			

^*^P-value < 0.05.

AEFI, adverse event following immunization.

## Discussion

We define adverse events following immunization (AEFI) as any untoward medical occurrence, which follows immunization that may be any unfavorable or unintended sign, abnormal laboratory finding, symptom, or disease (17). This study found that the prevalence of AEFI in the first dose vaccination is 16.7 and 22.6%, which is in line with the finding from the first and second phases of trials of CoronaVac (13). Meanwhile, another study in Pakistan found a higher rate of 33.5% after administering the first dose of inactivated COVID-19 vaccine Sinopharm (24). We found that the most common AEFI symptom was pain at the injection site, similar to the finding from the clinical phase study of the CoronaVac vaccine (13, 25) and other previous studies (24, 26). However, a high number of cough and common cold was found after the second dose of the COVID-19 vaccine. Although these two symptoms have been reported in phase clinical trials 1 and 2 of CoronaVac (27), it may also be a coincidence with COVID-19 infection. During the second dose vaccination period, the COVID-19 pandemic was entering the waves of omicron variants in Indonesia (28).

We also found some AEFI symptoms that are not stated on the manual vaccine of CoronaVac, such as sleepiness and hungry. Supangat et al. (29) reported that sleepiness was the second most common systemic effect among Indonesian medical clerkship students after receiving the CoronaVac vaccine. Another study by Franck et al. (30) found that sleep duration in the first 24 h after immunization was increased. Hendarto et al. (31) and Rachman et al. (32) found that feeling hungry is one of the AEFI symptoms reported by Indonesian CoronaVac recipients. Sleepiness may be explained by the immune response activated by the vaccine, such as stress-related modulation of cytokine production by activated T cells that may enhance an inflammatory response to the hypothalamus response to vaccination (33). Sleep duration after vaccination may influence the immune response and boost the virus-specific adaptive cellular immunity (34). A similar mechanism through activated immune response may explain hungry after immunization.

Some allergic reactions such as nausea and vomiting also appeared. These two symptoms are appropriate for non-anaphylactic allergic reactions that may be caused by non-human proteins, preservatives, or stabilizers in vaccine formulas (27). Symptoms of AEFI mostly appear on the same day after receiving the first dose and recover within 1–4 days. This finding is slightly different from findings of other COVID-19 inactivated virus vaccine that reports that many AEFIs occur in 1–7 days and recover in 2 days (13, 24, 35).

A study about Chad0x1 (AstraZeneca) as an activated-virus vaccine in children aged 6–17 years is slightly different with the loss of appetite symptoms, but the most frequent local symptoms are pain and tenderness. While in systemic symptoms, fatigue and headache were commonly reported. However, no severe symptoms found (36). Another study in the community also found the same results for the symptoms, although it was conducted in an older age (26, 35).

History of AEFI in childhood vaccination was significantly associated with the occurrence of AEFI after administering the first dose. This finding may be related to the manufacture of the vaccine itself, while some vaccines received by children when childhood have the same type as CoronaVac, which is inactivated virus (37). Especially, when children have a history of allergy to a vaccine component, it may increase the risk of AEFIs (38). Another study explained that a history of pain at site injection and fever after vaccination might increase the risk for recurrent AEFI with less or the same severity (37).

We found that children with a history of administering other vaccines within 1 month before the COVID-19 vaccination had a higher risk of having AEFI. This might be explained by the accumulation of aluminum adjuvant, which can trigger a local inflammatory reaction and less often causes systemic effects such as exacerbation of autoimmune diseases and allergies (18). One month before the administration of the COVID-19 vaccine, there was a school child immunization month (BIAS) program using diphtheria–tetanus (Dt) vaccine for students in grade 1 and tetanus–diphtheria (Td) vaccine for the students in grade 2 and 5 of elementary schools. Both vaccines are inactivated vaccines containing an aluminum adjuvant, which is needed to enhance the immune response.

WHO has recommended co-administering the COVID-19 vaccine with another vaccine with a minimum interval of 14 days, but there was a study about influenza vaccine co-administering with the COVID-19 vaccine increased the risk for AEFI (39, 40).

Mass-setting for a place of vaccination may be related to anxiety-related AEFIs that were reported by Loharikar et al. (41) can occur in individuals receiving vaccinations by seeing their friend whose fear of needles and experiencing pain. This stimulation may decrease heart rate and vasodilation, cerebral hyperfusion, and the worst is a temporary loss of consciousness. The finding in this study was the opposite, mass-setting has a lower risk for AEFI. This may be due to the safety perception that mass-setting with a large number of health workers or staff support allows children to easily access get treatment while they are experiencing an AEFI (42).

Females are related to a tendency to report AEFI more than males also found in another study in communities (25, 43, 44). Besides, Bae et al. (44) *hypothesize* this finding as differences in immunological response between females and males, but this finding should be investigated more in the future study.

### Strength and limitation

To the best of our knowledge, this study is the first of its kind to date where active surveillance of the COVID-19 vaccine was conducted, especially on children aged 6–11 years. We could not find any similar published study in the public domain until the data submission date. Our study will provide additional data regarding AEFI of COVID-19 in 6–11 aged children in a real setting. We selected the respondents using stratified random sampling that may represent the prevalence of AEFI in our setting. However, this study has some limitations. We did not conduct a causality assessment of AEFIs, so we could not assess if the AEFI was due to the vaccine reaction or other causes. We also did not assess the severity of AEFI. However, we assess if the children visit health providers for the event. A small number (9.9%) of children visited health providers but did not need hospitalization. This may reflect that most AEFIs were mild to moderate.

A possibility of recall bias may affect this study because we collected information on the fourth week after the dose. Children who developed serious AEFIs may not be covered in this study because they may not attend to get the second dose despite having a greater chance of experiencing recurrent AEFIs at dose two (45). Those who were experiencing non-serious AEFIs on the first dose could receive dose two with the same vaccine (46). Because the data collection was conducted at dose two vaccination, this study can only identify non-serious AEFIs.

Since this study has a limited participant population of children aged 6–11 years who may not reflect the general population demographic that used the CoronaVac vaccine, more extensive long-term studies with better representation of younger or older age groups are warranted. The higher occurrence of AEFIs in individuals with a history of AEFI in childhood vaccination needs to be investigated in future research.

## Conclusion

The prevalence of AEFI in the first dose and the second dose of inactivated COVID-19 vaccine was comparable to that reported in the clinical trial study. Risk communication should be provided to the child and their parents regarding the risk of mild AEFI of the COVID-19 vaccine, especially for children with a history of AEFI in childhood vaccination and who received other vaccines containing the same adjuvant with CoronaVac within 1 month. A mass-setting of vaccination should be taken as an advantage to educate parents about the risk of AEFI and also about the reporting pathways.

## Data availability statement

The original contributions presented in the study are included in the article/supplementary files, further inquiries can be directed to the corresponding author.

## Ethics statement

The studies involving human participants were reviewed and approved by the Ethics Committee of the Faculty of Medicine, Public Health, and Nursing, Universitas Gadjah Mada, with registration number KE/FK/0112/EC/2022. The participants legal guardian/next of kin provided their written informed consent to participate in this study.

## Author contributions

FP: concept, design, data collection, data analysis, first draft writing, and final draft writing. MS: concept, design, review, and edited the manuscript. RA: concept, design, and review of the manuscript. All authors contributed to the article and approved the submitted version.

## Funding

This study is supported by a grant from the Faculty of Medicine, Public Health, and Nursing, Gadjah Mada University, Indonesia.

## Conflict of interest

The authors declare that the research was conducted in the absence of any commercial or financial relationships that could be construed as a potential conflict of interest.

## Publisher's note

All claims expressed in this article are solely those of the authors and do not necessarily represent those of their affiliated organizations, or those of the publisher, the editors and the reviewers. Any product that may be evaluated in this article, or claim that may be made by its manufacturer, is not guaranteed or endorsed by the publisher.
